# Multimodal Management of Facial Acne Scarring Using Energy-Based Devices and Injectable Therapies: A Case Report

**DOI:** 10.7759/cureus.102151

**Published:** 2026-01-23

**Authors:** Luis L Velázquez Arenas, Sarahi Garay Enriquez, Daniela Gómez

**Affiliations:** 1 Dermatology, School of Medicine and Health Sciences at Tecnológico de Monterrey, Monterrey, MEX; 2 Medicine, School of Medicine and Health Sciences at Tecnológico de Monterrey, Monterrey, MEX

**Keywords:** acne scars, atrophic scars, erbium:yag laser, hyaluronic acid, picosecond laser, poly-l-lactic acid

## Abstract

Acne vulgaris is a highly prevalent chronic inflammatory disorder that frequently results in permanent scarring, with atrophic scars representing the predominant type. The management of atrophic acne scarring is challenging due to variability in scar morphology, depth of dermal involvement, and patient-specific factors, often necessitating individualized and multimodal treatment strategies. We report the case of a 35-year-old male with facial atrophic acne scars treated using a combination of picosecond laser therapy, erbium:YAG laser resurfacing, injectable poly-L-lactic acid, hyaluronic acid filler, and microneedling radiofrequency over a structured treatment course. The patient demonstrated progressive improvement in scar appearance and skin texture, with good tolerability and no reported adverse events throughout follow-up. This case highlights the clinical application of a multimodal approach that integrates energy-based devices and injectable therapies to address multiple structural components of atrophic acne scarring.

## Introduction

Acne vulgaris is a chronic inflammatory disorder of the pilosebaceous unit and remains one of the most prevalent dermatologic conditions worldwide, affecting approximately 9.4% of the global population and up to 93% of adolescents [1,2]. Its pathogenesis is multifactorial and involves androgen-mediated sebaceous gland activity, increased sebum production, abnormal follicular hyperkeratinization, proliferation of *Cutibacterium* acnes, and subsequent inflammatory immune responses [2]. Acne scarring is a common long-term sequela resulting from dermal injury during lesion resolution, affecting up to 50% of individuals with acne [3]. Several factors have been associated with an increased risk of scar formation, including male sex, positive family history, and greater disease severity [1]. Approximately 80%-90% of acne scars are atrophic in nature, reflecting a net loss of dermal collagen during inflammatory and reparative phases [4].

Atrophic acne scars are categorized into three morphologic subtypes: ice pick scars (60%-70%), boxcar scars (20%-30%), and rolling scars (15%-25%). This morphologic variability has direct implications for therapeutic decision-making. Current treatment options include surgical approaches, such as excision, subcision, and punch techniques, as well as nonsurgical modalities, including dermabrasion, microneedling, chemical peels, injectable fillers, and energy-based devices [4]. Here, we report a case of atrophic acne scarring managed using a multimodal approach incorporating picosecond laser technology, erbium:YAG laser resurfacing, injectable poly-L-lactic acid and hyaluronic acid (HA), and radiofrequency treatment, highlighting the importance of individualized assessment and treatment planning.

## Case presentation

**Figure 1:**
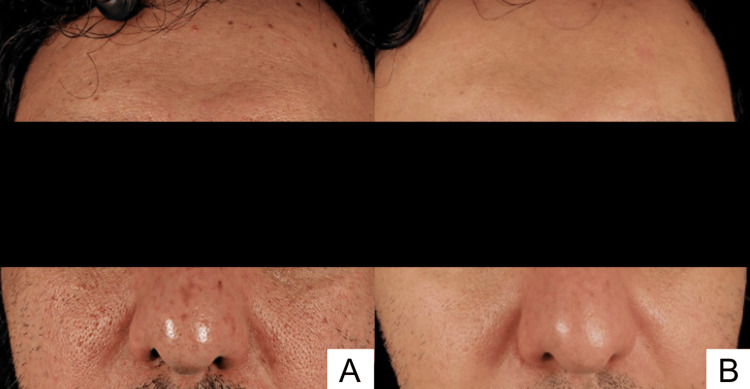
Clinical photographs demonstrating progressive improvement of acne scars over time. (A) Two months after the first treatment session (April 2024). (B) Prior to the final session (July 2025).

**Figure 2:**
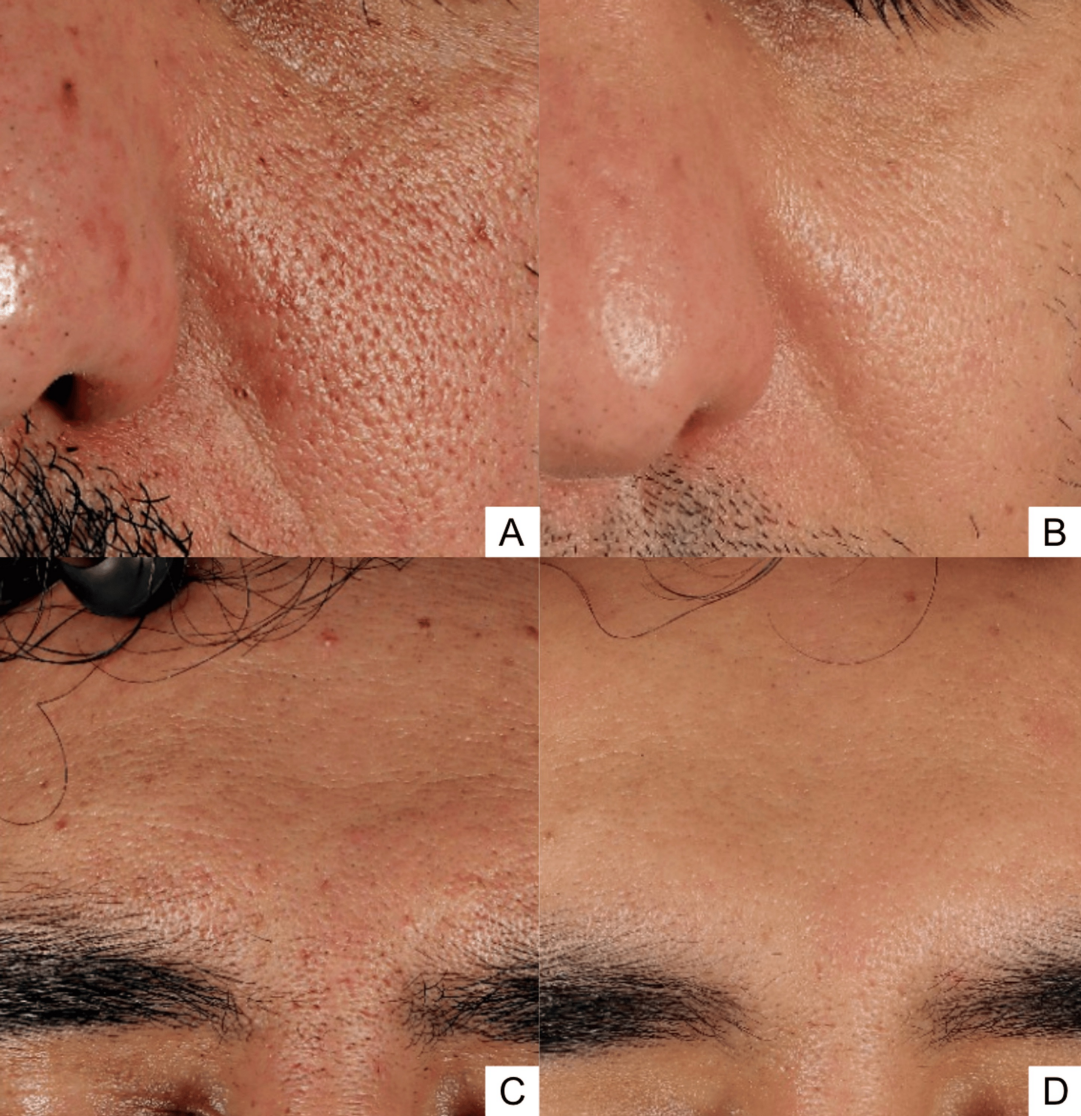
Representative close-up clinical photographs of the cheek (A, B) and frontal facial view (C, D). Images A and C were obtained in April 2024, whereas images B and D were obtained in July 2025, demonstrating improvement in skin texture and overall facial appearance.

A 35-year-old male with a history of inflammatory acne, previously managed with topical and systemic therapies, including oral antibiotics and isotretinoin, presented for the management of facial acne scars. The patient had no relevant medical comorbidities and no contraindications to treatments. On January 29, 2024, the patient underwent combined laser therapy for acne scarring and skin pigmentation using picosecond laser technology (Discovery Pico®) fractional 8 mm handpiece at an energy of 0.5 J and pulse frequency of 10 Hz, followed by Laser Erbium-Yag Resurfacing (Dermablate®) at resurfacing E 10% 20 J. The procedure was well tolerated, with no immediate or delayed adverse effects reported. As part of a comprehensive skin rejuvenation and scar remodeling protocol, additional treatments were performed over the following year. On February 26, 2025, poly-L-lactic acid (Sculptra®) was injected in the facial region to improve dermal thickness and collagen stimulation. This was followed by a second Sculptra session on April 28, 2025. On May 19, 2025, the patient received microneedling with radiofrequency treatment (Intensif®) in two facial areas to further enhance skin tightening and texture. Treatment parameters were individualized by anatomical area: the periocular region was treated with settings of 80 ms pulse duration, 10 W power, and 1.5 mm penetration depth; areas with pre-existing scars were treated at 110 ms, 15 W, with a depth of 3.0 mm; and areas without scars were treated at 110 ms, 14 W, with a depth of 2.5 mm. A third facial Sculptra session was performed on June 11, 2025, during which HA filler was also injected specifically into rolling-type acne scars. On June 24, 2025, a second combined laser session was carried out involving the face and neck. Treatment parameters included a picosecond laser using a fractional 8 mm handpiece at 10 Hz, with an energy of 0.7 J on the face and 0.6 J on the neck, followed by Er:YAG resurfacing, operating at E 10% 30 J. The patient reported satisfactory tolerance and uneventful recovery. Finally, on July 7, 2025, a second session of microneedling with radiofrequency treatment was performed on the face and neck. Throughout the treatment course, progressive improvement in acne scar appearance and skin texture was observed, with no adverse events reported (Figures 1A-1B, Figures 2A-2D).

## Discussion

Management of atrophic acne scarring remains clinically challenging due to the heterogeneity of scar morphology, depth of dermal involvement, and patient-specific factors, such as skin type and risk of postinflammatory hyperpigmentation (PIH) [5]. As no single modality adequately addresses all components of atrophic scarring, combination-based strategies are commonly incorporated into the treatment plan. Among energy-based devices, picosecond laser technology represents an important advancement in the treatment of atrophic acne scarring due to its ultrashort pulse duration and predominantly photoacoustic mechanism of action. These characteristics make it particularly suitable for patients with increased susceptibility to PIH or heightened sensitivity to pain [6]. Similarly, Kunzi-Rapp et al. evaluated the efficacy of Erbium:YAG laser therapy for acne scarring in a group of 12 patients, reporting an excellent response in 50% of cases, good in 25%, and fair in 25%, with no patients showing lack of improvement at three to six months of follow-up [7]. These outcomes are consistent with the clinical improvement observed in our patient, further supporting the effectiveness of Erbium:YAG laser therapy for acne scar management. Moreover, the comparative study by Rajput et al. demonstrated that fractional microneedling radiofrequency provides efficacy comparable to fractional CO₂ laser in the treatment of atrophic acne scars, achieving significant improvements in scar severity while offering the advantage of reduced downtime, lower incidence of PIH, and high patient satisfaction [8]. On the other hand, injectable fillers represent an established treatment modality for atrophic acne scarring, particularly rolling scars, with HA commonly used for localized correction and poly-L-lactic acid providing gradual volumization when injected into the subcutaneous plane. In addition, by stimulating neocollagenesis, poly-L-lactic acid contributes to progressive contour correction of depressed scars [9,10]. In our patient, HA was selectively injected into rolling-type scars, while poly-L-lactic acid was used to address diffuse dermal atrophy. Collectively, these interventions improve scar appearance by addressing distinct pathophysiologic mechanisms.

## Conclusions

In the present case, the integration of picosecond laser therapy, erbium:YAG laser resurfacing, injectable poly-L-lactic acid and HA, and radiofrequency treatment allowed for a comprehensive approach targeting multiple pathogenic components of atrophic acne scarring, including collagen loss, dermal atrophy, and textural irregularities. This multimodal strategy underscores the importance of individualized assessment and treatment planning based on scar subtype, distribution, and patient characteristics. Such an approach may optimize outcomes while minimizing adverse effects. While larger prospective studies are needed to further define optimal treatment sequencing and long-term outcomes, this case supports the role of combination-based interventions in the management of complex atrophic acne scars.
